# Charlin’S Syndrome Following a Routine Septorhinoplasty

**Published:** 2018-01

**Authors:** Ali Kavyani, Ali Manafi

**Affiliations:** 1 Department of Plastic Surgery, School of Medicine, Shiraz University of Medical Sciences, Namazi Hospital, Shiraz, Iran;; 2 Department of Plastic Surgery, School of Medicine, Iran University of Medical Sciences, Shiraz, Iran

**Keywords:** Septorhinoplasty, Complication, Charlin’s syndrome

## Abstract

There are some rare but probable devastating complications following any rhinoplasty. Charlin’s syndrome is a typical one. It is completely related to the external nasal nerve. In this report, we are presenting a 21-year-old female with signs and symptoms of Charlin’s syndrome, persisting for 4 years after a routine septorhinoplasty operation. Surgery was uneventful and the patient underwent bony septal resection and caudal septal relocation. Osteotomy was internal low to low and external transverse bilaterally. Overall, a routine septorhinoplasty was executed. Everything went well postoperatively, until 4 months after surgery, when some irritating symptoms developed and gradually intensified.

## INTRODUCTION

**Fig. 1:**
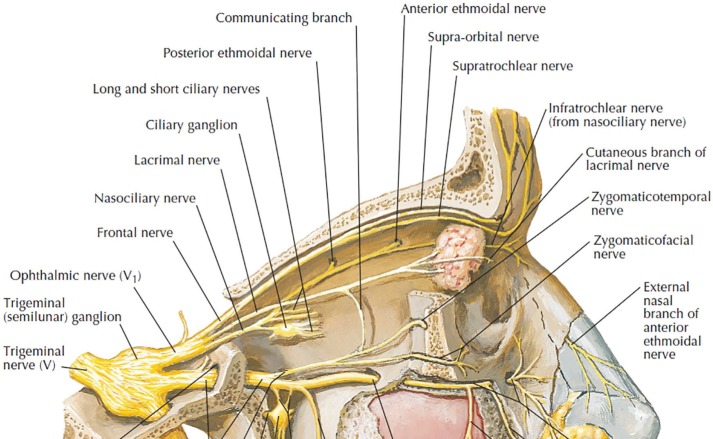
V1 branch of 5^th^ cranial nerve. (from Frank. H. Netter: Atlas of human anatomy. 6^th^ edition. Elsevier. 2014).

**Fig. 2:**
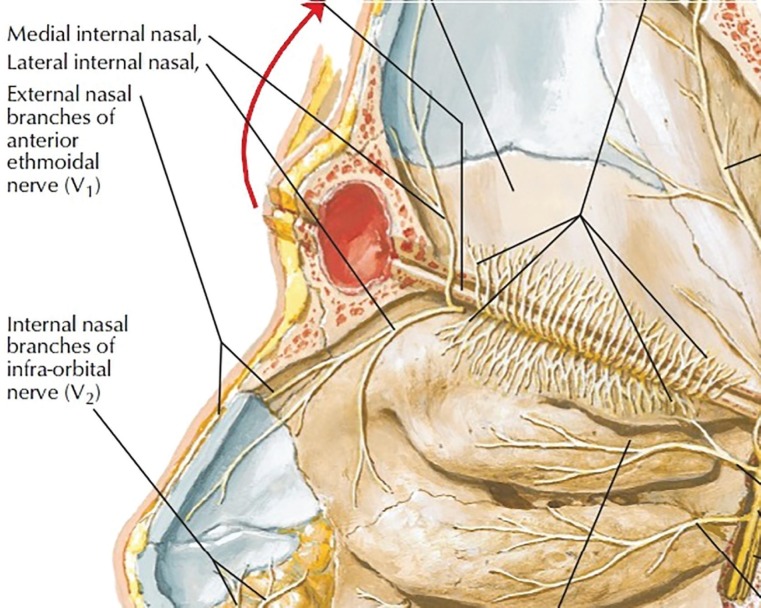
Anterior ethmoidal nerve. (from Frank. H. Netter: Atlas of human anatomy. 6^th^ edition. Elsevier. 2014).

Nasal envelope sensory innervation is principally derived from V1 (ophthalmic) and V2 (maxillary) branches of trigeminal (5^th^ cranial) nerve. Nasociliary nerve (a branch of V1) gives rise to anterior ethmoidal and finally infratrochlear nerve (Figure 1 and 2).^1^ Anterior ethmoidal nerve has two main branches: external and internal nasal nerves. The external nasal nerve exits through the lateral junction between upper lateral cartilage and nasal bone, on each side. Then, it travels through the nasal envelope to supply sensation for ipsilateral ala and sidewall. On occasion, it provides sensation to the tip and even the eye.^2^^,^^3^

According to the illustrations and anatomic descriptions above, the external nasal nerve exits through the lateral junction between upper lateral cartilages and nasal bones bilaterally. At this point, there is mobile cartilage, but stable and static bone. The shearing force between two different anatomic structures can irritate or damage the nerve.^4^^,^^5^ In rhinoplasty, this nerve is commonly injured or transected during skletonization or osteotomy. Most often, the only sequela is mild and trivial hypoesthesia of the tip or lateral sides of the nose. Although rare, a painful neuroma of the nerve stump, results in devastating symptoms of headache including upper facial and ocular pain, severe ipsilateral rhinorrhea and mucosal congestion, conjunctival injection. These symptoms and signs are called Charlin`s syndrome.^6^

## CASE REPORT

**Fig. 3:**
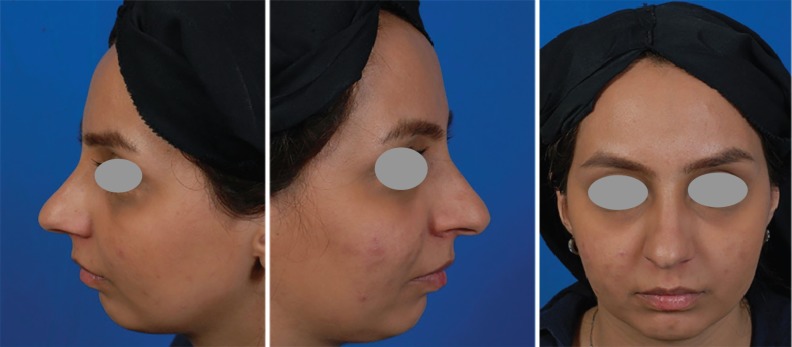
Preoperative photos. She refused a simultaneous genioplasty.

**Fig. 4:**
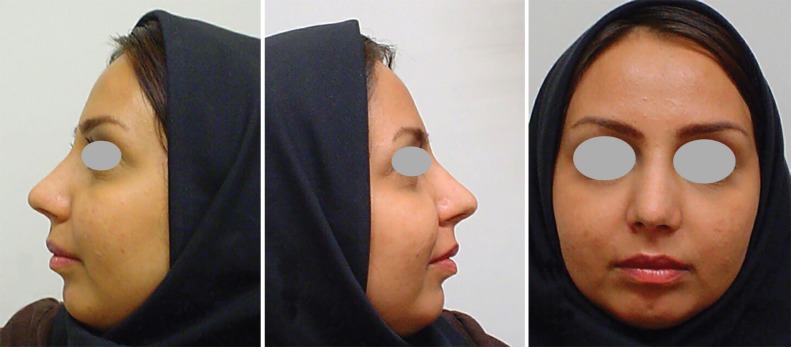
1 month postoperative photos.

A 21-year-old female underwent a routine septorhinoplasty for aesthetic and airway improvement. After envelope elevation in the sub-SMAS plane and total septal exposure through sub-perichondrial route, she underwent deviated bony and cartilaginous septal resection and caudal septal relocation, saving a sufficient L-strut. Osteotomy was internal low to low and external transverse bilaterally. Midvault reconstruction using bilateral spreader grafts was performed. Tip refinement was done by routine sutures and columellar strut graft was inserted. No tip graft was used. Overall, a routine septorhinoplasty was executed (Figure 3 and 4).

There was not any problem during postoperative period, but 4 months later, she gradually developed paroxysms of headaches followed by abundant right sided rhinorrhea. Periods of headache were felt once weekly, localized in the nasal root and right retro-ocular area and lasted about 30-45 minutes. Bilateral conjunctival injection (worse in the right side), was an accompanying symptom. All symptoms and signs aggravated gradually, eventually interfering with normal life.

**Fig. 5:**
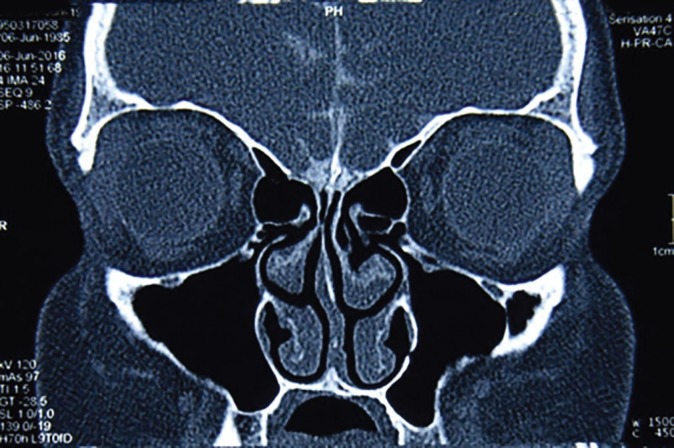
Postoperative coronal CT of th nasal area. Caudal septal relocation and bilateral open airway are evident.

**Fig. 6:**
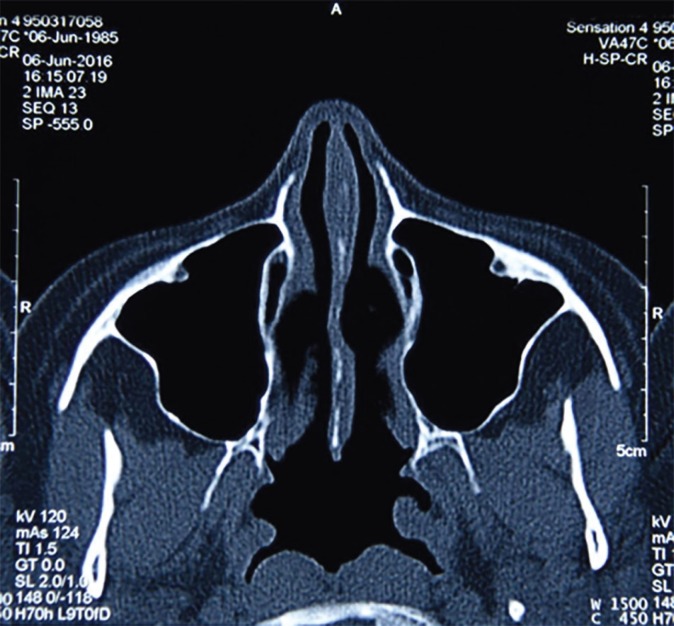
Postoperative axial CT shows corrected septal deviation, open airway, and almost normal view of the area.

At first, a list of differential diagnoses including infection, delayed retro-ocular hematoma or pseudoaneurysm, cavernous sinus thrombosis, different types of headaches and neuralgias, and intracranial pathology was arranged. Multiple studies including CT cysternography, dacrocystography, brain MRI, ophthalmologic evaluation and intraocular pressure monitoring were accomplished and all were normal. There was no problem in the routine hematologic and blood chemistry tests. Rhinorrhea liquid was examined clinically and pathologically to rule out CSF leakage that was negative. It was quite clear and non-purulent (Figure 5 and 6).

After failure to diagnose the original cause of symptoms and signs, the patient was referred to a neurologist. Motor and cranial nerve examinations were normal, with no finding in favor of central or peripheral demyelinating disorders. MRI showed no intracranial pathology. Cluster headache, trigeminal neuralgia and temporal arteritis were all ruled out. Ophthalmologic, optometric, perimetric and lacrimal system examinations were normal. There was no past medical history, except depression and anxiety, appropriately treated by sertraline, clomipramine and propranolol.

**Fig. 7:**
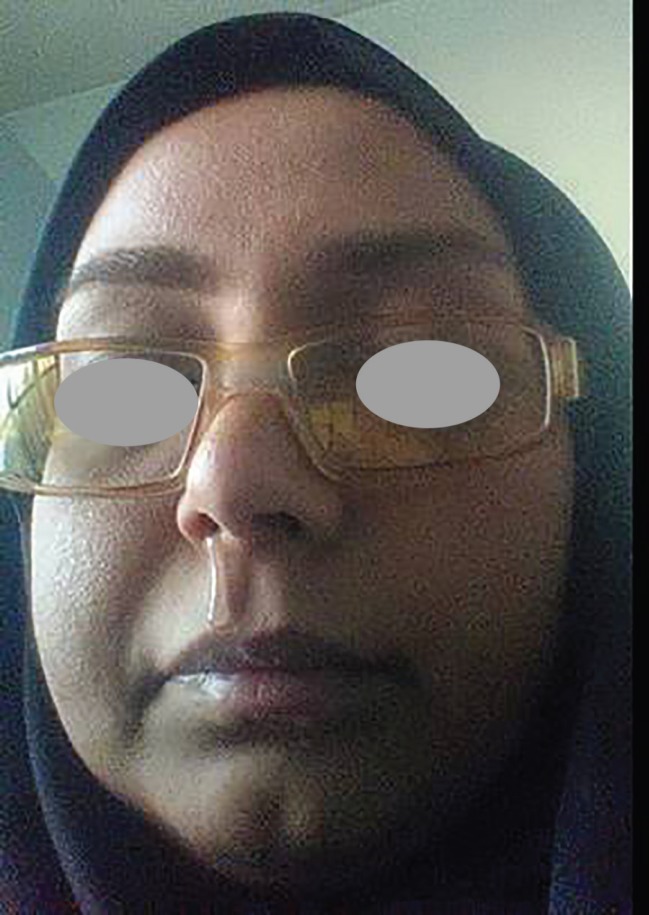
The patient with her chief complaint: persistent unilateral rhinorrhea.

The principal distressing problem of the patient was abundant and uncontrollable runny nose. She was pain free between headache attacks, but rhinorrhea was the persistent symptom and sustained during daily activities. It was very annoying and did not respond to any kind of medical therapy including H1 blockers and local or systemic decongestants. A comprehensive review of the literature was planned. There were few papers or textbooks explaining these symptoms. Among rare complications after rhinoplasty, we encountered the Charlin`s syndrome! Literature content regarding this rare syndrome was extremely scarce. The first session of nerve block with a local anesthetic in the area around the nasal bones, confirmed the diagnosis (Figure 7). 

To treat the patient, authors decided to re-operate and explore surgically, however, the patient refused a revision surgery. So, phenol solution was used to ablate the irritating nerve ending. Symptoms and signs were alleviated but remained after two sessions of injection. Phenol was injected to the area around the nasal bones. During the third session, phenol was injected to the area beneath the nasal bones, bilaterally. Just after 2-3 weeks, patient`s pain was eradicated but rhinorrhea persisted and it took several months to stop completely. 

## DISCUSSION

Charlin`s syndrome is a diagnosis of exclusions. Some important differential diagnoses including intracranial or retro-orbital tumors, demyelinating disorders, chronic paroxysmal hemicrania, trigeminal neuralgia, temporal arteritis and cluster headache should be ruled out. So, neurologic or neurosurgical consultation is mandatory.^7^ Headache in Charlin`s syndrome resembles cluster headache (sphenopalatine ganglion neuralgia), in which retro-orbital and frontal pain starts suddenly, peaks rapidly with typical short periods (45-60 minutes). Some different features are no seasonal or chronobiologic pattern and absence of alcohol-induced attacks in Charlin`s syndrome.^8^

**Fig. 8:**
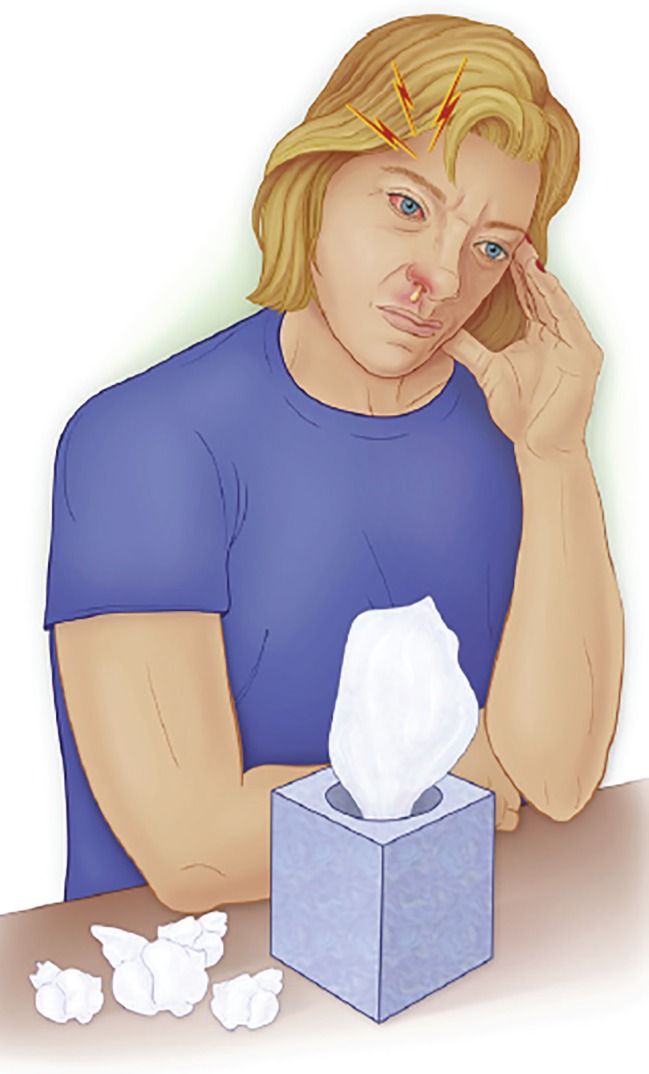
Patients suffering from Charlin’s syndrome present with the complaint of severe paroxysms of ocular or retro-orbital pain that radiates into the ipsilateral forehead, nose, and maxillary region. The pain is associated with voluminous ipsilateral rhinorrhea and congestion of the nasal mucosa and significant inflammation of the affected eye (from Waldman SD: Atlas of uncommon pain syndromes. 3rd ed., Elsevier 2013).

Precise neurologic and ophthalmologic studies, including accurate examination and appropriate imaging are pre-requisites for diagnosis. The cornerstone of diagnosis is having Charlin`s syndrome in mind (Figure 8). 

The diagnosis is confirmed by application of a local anesthetic to block external nasal nerve (by injection into the nose), anterior ethmoidal nerve (with intranasal applicator) or nasociliary nerve (by injection through medial orbital route), during paroxysms of headache. The pain will subside or eliminate in minutes, but temporarily.^1^^,^^2^ The treatment of Charlin’s syndrome is analogous to the treatment of trigeminal neuralgia. The use of anticonvulsants such as carbamazepine and gabapentin represents a reasonable starting point. High-dose steroids tapered over 10 days also have been anecdotally reported to provide relief. For patients who do not respond to the previously mentioned treatments, daily nasociliary ganglion block with local anesthetic and steroid is a reasonable next step.^8^

The definitive and permanent therapy for Charlin`s syndrome is chemical ablation or surgical transection of the nasociliary or anterior ethmoidal nerves.^1^^,^^2^ Anterior ethmoidal nerve is approached trans-nasally but nasociliary nerve is accessed through medial orbital cavity.^9^ Greater Auricular Nerve Block for Charlin`s Syndrome has been stated by some authors;^10^ but, the theoretical basis behind it remains a matter of debate.

## References

[1] Sluder G (1922). Nasociliary neuralgia. Ann Otolaryngol.

[2] Littel JJ (1946). Disturbances of the ethmoid branches of the ophthalmic nerve. Arch Otolaryngol.

[3] Sluder G (1927). Nasal Neurology, Headaches and Eye Disorders.

[4] Golding-Wood DG, Brookes GB (1991). Post-traumatic external nasal neuralgia-an often missed cause of facial pain?. Posgrad Med J.

[5] Rozen T (2009). Post-traumatic external nasal pain syndrome (a trigeminal based pain disorder). Headache.

[6] Becker M, Kohler R, Vargas MI, Viallon M, Delavelle J (2008). Pathology of the trigeminal nerve. Neuroimaging Clin N Am.

[7] Lewis DW, Gozzo YF, Avner MT (2005). The “other” primary headaches in children and adolescents. Pediatr Neurol.

[8] Waldman SD (2013). Atlas of uncommon pain syndromes.

[9] Waldman SD (2017). Atlas of pain management injection techniques.

[10] Waldman SD (2017). Atlas of pain management injection techniques.

